# Bilateral Knee Arthroplasty in Patients Affected by Windswept Deformity: A Systematic Review

**DOI:** 10.3390/jcm11216580

**Published:** 2022-11-06

**Authors:** Eugenio Cammisa, Iacopo Sassoli, Matteo La Verde, Stefano Fratini, Vito Gaetano Rinaldi, Giada Lullini, Vittorio Vaccari, Stefano Zaffagnini, Giulio Maria Marcheggiani Muccioli

**Affiliations:** 1 Orthopedic Unit, Imola Hospital, 40026 Imola, Italy; 2 II Orthopaedic and Traumatology Clinic, IRCCS Istituto Ortopedico Rizzoli—DIBINEM, University of Bologna, Via di Barbiano, 1/10, 40100 Bologna, Italy; 3 UOC Medicina Riabilitativa e Neuroriabilitazione, IRCCS Istituto delle Scienze Neurologiche di Bologna, 40139 Bologna, Italy

**Keywords:** knee arthroplasty, TKA, windswept, varus knee, valgus knee, kinematic alignment

## Abstract

Background: “Windswept” deformity (WSD) consists of a non-frequent condition in which the patient presents a valgus deformity in one knee and a varus deformity in the other. We performed a review of the available literature to aggregate the accessible data on the outcomes of bilateral knee arthroplasty in patients with WSD and to discuss the surgical challenges that this condition might pose. Methods: A systematic review of the literature following the PRISMA guidelines was conducted. The relevant studies between 1979 and 2021 were identified. Four studies with a total of 68 patients were included for analysis. The mean follow-up for varus knees was 3.3 years, 3.1 years for valgus knees. The quality and rigor of the included studies was assessed using the Methodological index for non-randomized studies (MINORS). Results: All the studies reported improvement in knee function following knee replacement surgery, and a reduction in axial deviation of both knees, with similar results in valgus and varus knees in terms of patient satisfaction. The most relevant data were that unicompartmental knee arthroplasty (UKA) allowed for limited axial correction with slightly inferior functional results. Kinematic alignment (KA) allowed for similar results in both knees. Conclusion: The present review shows how satisfactory results can be achieved in both knees in patients with WSD and osteoarthrosis (OA). However, the operating surgeon should be aware of the importance of the implant choice in terms of functional outcomes. In the absence of extra-articular deformities, calipered KA total knee arthroplasty (TKA) can be performed on both knees with good axial correction and functional outcome. Level of evidence: II —Systematic review of cohort studies.

## 1. Introduction

“Windswept” deformity (WSD) consists of a non-frequent condition in which the patient presents a valgus deformity in one knee and a varus deformity in the other. These concomitant deformities present some unique challenges when performing knee arthroplasty on these patients [1].

The patients with WSD knees have opposite deformities in the coronal plane, and each knee may present different insufficiencies of bone and soft tissue [2]. Windswept deformities (WSD) can be recognized on anterior-posterior (AP) radiography, where the alignment of the knees can be obtained with the use of the hip-knee-ankle angle (HKA) or the femorotibial angle (FTA), and an opposite axial deviation is present in the same patient [3].

The underlying cause of WSD can vary. It often correlates with skeletal dysplasia, physeal disturbances, metabolic bone disorders, rheumatic arthritis, and post-trauma, while it remains an unusual condition in patients with primary arthritis [4].

In WSD, medial compartment osteoarthritis (OA) on the side of varus deformity and lateral compartment OA on the side of the valgus deformity can be found. There is also a contracture of the soft tissues on the medial side of the knee, which often need to be released in varus knees to achieve satisfactory results. The same is true for the lateral ligaments and soft tissues in valgus knees [5,6]. Other crucial challenges are the different degrees of patellofemoral arthritis and the patellar tracking that should be optimized to gain superior outcomes: This is especially important in valgus knees, which can require an extensive lateral retinacular release. In bilateral OA in WSD, knee arthroplasty can be performed in one or two stages, and the choice depends on the clinical criteria of the patient and the will of the surgeon [7,8].

In literature, few studies analyzed windswept deformities and their outcomes after bilateral knee arthroplasty.

The purpose of our study is to perform a review of the available literature to aggregate the accessible data on the outcomes of bilateral knee arthroplasty in patients with WSD and to discuss the surgical challenges that this condition might pose to the surgeon.

## 2. Materials and Methods

### 2.1. Search Strategy

The literature search was operated on Medline, EMBASE and Cochrane CENTRAL on 20 September 2022 by two researchers. The string used for the search was: “(windswept OR (valgus AND varus)) AND (tka OR ((‘arthroplasty’/exp OR arthroplasty) AND (‘knee’/exp OR knee)) OR ((‘total’/exp OR total) AND (‘knee’/exp OR knee))) AND bilateral”.

All relevant studies were identified in accordance with the Preferred Reporting Items for Systematic review and Meta-Analysis (PRISMA) guidelines (Figure S1). The authors also evaluated the references of the included articles, so it was possible to trace a further study that was added to our review as it met all the inclusion and exclusion criteria. The selected articles adhered to the systematic reviews’ Population, Intervention, Comparison and Outcomes (PICO) criteria. The review was registered on the International prospective register of systematic reviews (PROSPERO) with the following registration number: CRD42022361781.

### 2.2. Eligibility Criteria

Our inclusion criteria for the study were as follows: (1) the studies about knee arthroplasty as a treatment modality in WSD, (2) the patients of each article with WSD must have been treated with arthroplasty in both knees, (3) the articles should report functional outcomes before and after knee arthroplasty.

Our exclusion criteria were: (1) articles not reporting on the functional outcomes, (2) articles not reporting on the preoperative and postoperative knee alignment, and (3) articles that included other treatments for WSD instead of knee arthroplasty. After the removal of duplicate articles, a full-text review of the selected studies was undertaken by two independent junior authors (IS and MLV).

### 2.3. Data Extraction

The data were extrapolated from the selected documents using a standardized data collection form. Information on the number of patients, their demographic data, the follow-up period, the type of implant, and if the arthroplasty was made in one stage or two stages were reported in a spreadsheet. In order to simplify data collection and facilitate readability, the preoperative and postoperative clinical and functional outcomes were compiled into two spreadsheets, one for the preoperative valgus knee and one for the preoperative varus knee. There were no inconsistencies in the results.

### 2.4. Quality Assessment and Risk of Bias

Two authors assessed the quality and rigor of the included studies using the Methodological Index for Non-randomized Studies (MINORS) [9]. The global ideal score is 16 for non-comparative studies. The items were scored 0 if not reported; 1 when reported but inadequate; and 2 when reported and adequate. Consensus was reached by the two reviewers (IS/MLV) when there was no difference in opinion on an item. If no consensus was reached, the independent opinion of a third reviewer was decisive (EC). The individual scores are reported in Table 1.

### 2.5. Statistical Analysis

The results were summarized using descriptive statistics for continuous variables, frequencies, and percentages for categorical variables. Microsoft Excel, 2016 version (Microsoft Corporation, Redmond, WA, USA) was used for data analysis.

## 3. Results

### 3.1. Search Results

Initially, the search identified 128 articles, 38 of which were duplicates. Of the remaining 91 articles, 65 were eliminated because they did not fit the study’s inclusion criteria.

From the remaining 26 articles, two were not included because it was not possible to retrieve the complete text. At the same time, another 20 were eliminated after a full-text analysis for the following reasons: patients included in the study did not present WSD, the patients did not undergo bilateral knee arthroplasty, patients were not treated with arthroplasty, articles that presented only the abstracts, articles without any score measuring the clinical outcome.

All four studies met the inclusion criteria and reported clinical and functional outcome scores to evaluate the treatment results and preoperative and postoperative knee alignment.

Population data and additional relevant data are included in Table 2.

The results are summarized in Table 3 for the valgus knees and Table 4 for the varus knees.

Each study included in the review was a retrospective case series of patients affected by windswept deformity of the knee and treated with joint replacement surgeries, for a total of 68 patients and 136 knees.

Three studies were considered at low risk of bias (MINORS higher than or equal to 14), one was considered at high risk of bias. The four studies included were all retrospective case series. The biggest one was Meding et al. [10] with 22 patients. The mean follow-up was at least two years for every study.

In three papers, total knee replacement was employed. Meding et al. [10] used cruciate-retaining (CR) implants, Song et al. [11] used a posterior stabilized (PS) design, while Howell et al. [12] used both designs with a prevalence of CR (79% of valgus knees, 89% of varus knees).

Tanaka et al. [13] only used unicompartmental knee implants for treating both types of deformity.

The follow-up time was greater than two years for all studies.

Only one author, Howell et al. [12], performed all the surgeries in a staged fashion. In 11 patients (58%), the valgus side was addressed first. All other authors reported either bilateral one-stage procedures or a combination of the two.

Howell et al. [12] performed the arthroplasties using a calipered kinematic alignment (KA), while the other authors used a mechanical alignment (MA).

Meding et al. [10] and Song et al. [11] described the necessity for ligament releases in some patients, summarized in Table 2.

Howell et al. [12] performed patellar resurfacing in all patients. Tanaka et al. [13] and Song et al. [11] do not mention any patellar treatment. Meding et al. [10] performed two patellar resurfacing procedures with metal backed component in the same patient, both required a revision.

### 3.2. Outcome Analysis

All authors reported increased clinical and functional scores after surgery; the individual scores are reported in Table 3 and Table 4.

Meding et al. [10] and Tanaka et al. [13] evaluated the range of motion in terms of flexion and extension of the knee before surgery and at follow-up. Although there was no significant difference in the flexion range after surgery, hyperextension was reduced after the surgery in varus and valgus knees in both sides.

All the studies in the review reported alignment through the accurate calculation of the FTA or the HKA before and after surgery; each study describes angles of both the varus and valgus knee.

The most significant alignment changes in the varus knees were reported by Song et al. [11], which went from a mean varus alignment of 7.8 ± 6.7 degrees (measured with FTA) to a mean valgus alignment of 5.4 ± 3.2, for an overall mean change of 13.2 degrees.

Regarding the valgus knees, each study demonstrated a more significant correction than the paired varus knees; Howell et al. [12] reported a mean correction of more than 10 degrees to a mean after surgery of 1° ± 2.3° (calculated with HKA angle).

Tanaka et al. [13] reported one case of deep-vessel thrombosis, which was fully treated with antithrombotic therapy. One case of radiolucency under the tibial component at follow-up was also reported and treated conservatively. Meding et al. [10] reported in the varus group one case of patella revision, one case of TKA revision, and one case of patella subluxation. In the valgus group, there was one case of patella revision and one superficial infection.

## 4. Discussion

The most important finding of this systematic review was that there was no significant difference in paired knees’ outcomes.

As is known, initial valgus and varus deformity affects the difficulty of TKA. Both bone tissue deformities and soft tissue imbalances concur to determine the success of the knee alignment correction [10]. Recently, Baldini et al. also demonstrated that contralateral limb alignment could affect the operated side, especially in great preoperative deformities. That is supposedly due to the adduction moment in association with extensive releases [14].

There is growing interest in the KA technique as an alternative to mechanical alignment (MA) for small and big deformities. KA allows the preservation of the native knee kinematics in minor deformities without affecting the ligament balance. KA is a more difficult choice in more significant deformities due to compromised ligaments and the increased risk of mechanical failure if an insufficient constraint is chosen. However, it is certainly interesting that in 19 patients with both varus and valgus deformity, Howell et al. [12] found no differences in the OKS and FJS between the paired knees at a mean follow-up of 2.3 years, and better results than some reported for MA TKAs [15].

Two studies in this review analyzed MA TKA. All the paired knee results were similar. A slight greater improvement from baseline is shown for varus knees due to lower PROMs scores reported before surgery. This difference is, however, well within the confidence intervals.

It must be noted that KA can correct for intra-articular deformities, while any extra-articular deformity remains unaddressed. Because of that, the authors suggest a careful study of the deformity when choosing KA over MA.

Since the WSD is a bilateral deformity often requiring bilateral surgical intervention, all the considerations usually made for bilateral TKA apply. The comparison between simultaneous (one-stage) and staged (two-stage) TKA is difficult because surgical indications for a one-stage procedure are mostly given to younger and healthier patients [16]. This represents a selection bias, which limits most of the studies in literature. What is currently known is that one-stage TKA costs less and has better rehabilitation outcomes than two-stage. The single hospitalization and physical therapy cycles are cheaper because of the shorter length of stay, but due to the double surgical trauma, the patient would need twice as many blood transfusions [17]. Moreover, Richardson et al. found higher mechanical complications and infection rates in two-stage TKA [18]. This could be explained by an orthopedic, mechanical concept: knee osteoarthritis is often associated with axial deformities and limb shortening [19], so their simultaneous correction maintains lower limb equal length, preventing pelvis and spine asymmetry imbalance. Patients’ selection could represent a limit because of different comorbidities between the two cohorts, as explained before. The indication must be given in function of each patient [20] and requires the approval of the colleague anesthesiologists. In both orthopedic and anesthesiologist complex cases, the choice should be two-stage, so it was in three of the WSD studies we included in this review.

Howell et al. [12] analyzed a cohort of 19 patients who underwent two-stage KA-TKAs, finding no differences in the OKS and FJS between paired knees with varus and valgus deformity at a mean follow-up of 2.3 years, and better results than MA-TKA [15].

Song et al. [11] studied a cohort composed of both simultaneous and staged TKAs for a total of 14 patients, eight of whom presented degenerative scoliosis associated with knee deformity. They found satisfactory clinical outcomes and overall patient satisfaction regarding pain relief and function, with no differences between kind of deformity and postoperative result.

Meding et al. [10] especially analyzed simultaneous bilateral TKAs (20 patients of 22), finding no differences between clinical outcomes in the varus and valgus groups postoperatively. Patients noted no side-to-side differences concerning pain or function at the final follow-up.

The cohort of Tanaka et al. [13] consisted of 13 patients subjected to bilateral one-staged unicompartmental knee arthroplasty. Two considerations must be made: the knees had smaller axial deformities compared to the other studies, and the postoperative OKS scores are significantly lower than what Howell et al. [12] reported. This is to be expected since UKA allows for smaller axial corrections.

Given these results, the authors suggest caution when choosing UKA in patients with WSD; even in carefully selected patients, the achieved results can be less than optimal.

Two studies reported on the ligament releases performed during surgery. It can be noted that valgus knees required more ligament releases to be correctly balanced. Deep medial collateral ligament releases for tight medial compartment, iliotibial band release, posterior capsule release and lateral patellar retinaculum release were all more frequent in valgus knee.

This review has several limitations. Firstly, the small amount of literature available on this rare condition makes the collectible data relatively unabundant. Only four studies for a total of 68 patients and 136 knees had met the inclusion criteria.

WSD is a rare condition not often found in clinical practice, and in the literature there is a lack of studies with larger sample sizes. Two studies did not fully report the pre- and postoperative data, posing a risk of bias. This furthermore highlights the importance of review studies to better compile and interpret all the available data from different sources.

The heterogeneity of treatment choices, such as implant design and prosthesis alignment, between the available studies hindered the possibility of a quantitative aggregate analysis. As a result, a meta-analysis was not performed. Nevertheless, the comprehensive comparative analysis of the results of the studies presented provides a good scope on the subject and a good starting point for further discussion. More studies on the subject are needed.

## 5. Conclusions

The present review shows how knee arthroplasty can achieve satisfactory results in both knees in patients with WSD and OA. However, the operating surgeon should be aware of the importance of the implant choice in terms of functional outcomes, given that UKA functional outcomes were inferior to TKA. In the absence of extra-articular deformities, calipered KA TKA can be performed on both knees with good axial correction and functional outcome.

## Figures and Tables

**Table 1:** Individual MINORS score.

	MINORS Criteria	Howell et al. [10]	Song et al. [11]	Meding et al. [12]	Tanaka et al. [13]
1	A clearly stated aim	2	2	2	2
2	Inclusion of consecutive patients	2	2	2	2
3	Prospective collection of data	2	1	2	2
4	Endpoints appropriate to the aim of the study	2	1	2	2
5	Unbiased assessment of the study endpoint	0	0	1	2
6	Follow-up period appropriate to the aim of the study	2	2	2	2
7	Loss to follow up less than 5%	2	1	1	2
8	Prospective calculation of the study size	2	2	2	2
9	An adequate control group				
10	Contemporary groups				
11	Baseline equivalence of groups				
12	Adequate statistical analyses				
	TOT	14	11	14	16

**Table 2:** TKA: Total Knee Arthroplasty; CR: Cruciate Retaining; PS: Posterior Stabilized; MA: Mechanical Aligned; KA: Kinematic Aligned; DVT: Deep Vessel Thrombosis, dMCL: deep Medial Collateral Ligament, PC: Posterior Capsule, LPR: Lateral Patellar Retinalculum, ITB: Ileotibial Band.

	Year	Journal	N. Patients	Implant Used	Simultaneous Bilateral Procedures	Two Stage Procedures	MINORS	Surgery Associated Releases	Complications
Howell et al. [10]	2019	KSSTA	19	Varus; 17 TKA CR KA, 2 TKA PS KA Valgus: 15 TKA CR KA, 4 TKA PS KA	0	19	14/16		
Song et al. [11]	2008	Kor Knee Surg	14	PS TKA MA	3	11 (within 7 days)	11/16	Varus: 11 dMCL, 2 PC, 1 ITB Valgus: 14 dMCL, 5 PC, 4 ITB	Valgus: 1 patellar clunk syndrome
Meding et al. [12]	2000	Jour Arthrop	22	TKA CR	22	0	14/16	Varus: 19 dMCL, 9 PC, 6 LPR Valgus: 22 dMCL, 9PC, 3 ITB, 8 LPR	Varus: 1 patella revision, 1 TKA revision, 1 patella subluxation Valgus: 1 patella revision, 1 superficial wound infection
Tanaka et al. [13]	2020	The Knee	13	UKA	13	0			1 DVT

**Table 3:** Valgus knees.

Authors	Age at Surgery (y)	FU (y)	Knee Motion			PROMS			Valgus Alignment Pre-op	Valgus Alignment Post-op
				Pre-op	Post-op		Pre-op	Post-op		
Howell et al. [12]	68 ± 7	2.3 (range 1–4)	Flexion (°)	114 ± 6	NA	KSS	34 ± 15	NA	11 ± 5 *	1 ± 2.3 *
			Extension (°)	10 ± 8	NA	FJS	NA	90 [69–96] **		
						OKS	24 ± 8	47 [39–47] **		
Song et al. [11]	66 ± 6.3	2.1 ± 0.9	Flexion (°)	NA	NA	HSS	64.7 ± 5.8	86 ± 4.9	10.21± 5.8	6.8 ± 1.4
			Extension (°)	NA	NA					
Meding et al. [10]	72.5 ± 9.32	5.6 ± 3.8	Flexion (°)	111.41 ± 11.97	111.5 ± 18	KSS	45.81 ± 18.70	87.64 ± 6.47	15.95 ± 5.88	6.09 ± 1.93
			Extension (°)	−5.23 ± 9.82	0.95 ± 2.59	KSS	40.68 ± 16.21	72.95 ± 17.23		
Tanaka et al. [13]	73 ± 7.8	2.6 ± 1.4	Flexion (°)	137 ± 10.7	132.9 ± 9.4	OKS	25.3 ± 9.7	34.9 ± 5.8	8.3 ± 5.3	4.3 ± 3.4
			Extension (°)	−3.8 ± 4.8	−1.7 ± 3.3					

KSS: Knee Society Score; OKS: Oxford Knee Score; FJS: Forgotten Joint Score; HSS: Hospital for Special Surgery knee score; NA: Not Available. If not otherwise specified, varus alignment is measured on standing anteroposterior radiograph as a deviation of the FTA from 180°. If not otherwise specified, values are expressed as mean and standard deviation. * measured on HKA. ** median and interquartile range.

**Table 4:** Varus knees.

Authors	Age at Surgery (y)	FU (y)	Knee Motion			PROMS			Varus Alignment Pre-op	Varus Alignment Post-op
				Pre-op	Post-op		Pre-op	Post-op		
Howell et al. [12]	68 ± 7	2.3 (range 1–4)	Flexion (°)	112 ± 5	NA	KSS	30 ± 11	NA	3 ± 2 *	0 ± 2.1*
			Extension (°)	10 ± 8	NA	FJS	NA	90 [73–100] **		
						OKS	22 ± 8	47 [41–47] **		
Song et al. [11]	66 ± 6.3	2.1 ± 0.9	Flexion (°)	NA	NA	HSS	61.5 ± 5.9	86.9 ± 5.24	7.8 ± 6.7	−5.4 ± 3.2
			Extension (°)	NA	NA					
Meding et al. [10]	72.5 ± 9.32	5.6 ± 3.8	Flexion (°)	109.77 ± 14.60	110.59 ± 9.91	KSS	37.59 ± 18.05	86.09 ± 9.07	5.90 ± 5.29	−5.91 ± 4.62
			Extension (°)	−5.23 ± 14.01	0.45 ± 2.13	KSS	40.23 ± 15.7	73.86 ± 17		
Tanaka et al. [13]	73 ± 7.8	2.6 ± 1.4	Flexion (°)	133.5 ± 17.7	130.00 ± 7.7	OKS	25.3 ± 9.7	34.9 ± 5.8	0.6 ± 5.1	−1 ± 4.2
			Extension (°)	−4.6 ± 4.8	−1.3 ± 2.3					

KSS: Knee Society Score; OKS: Oxford Knee Score; FJS: Forgotten Joint Score; HSS: Hospital for Special Surgery knee score; NA: Not Available. If not otherwise specified, varus alignment is measured on standing anteroposterior radiograph as a deviation of the FTA from 180°. If not otherwise specified, values are expressed as mean and standard deviation. * measured on HKA. ** median and interquartile range.

## Data Availability

The data presented in this study are available in the tables of this article.

## Supplementary Materials

The following supporting information can be downloaded at: https://www.mdpi.com/article/10.3390/jcm11216580/s1 Figure S1: PRISMA Flowchart.
