# Supplementary material for: Bilateral Knee Arthroplasty in Patients Affected by Windswept Deformity: A Systematic Review
Source: J Clin Med. 2022 Nov 6;11(21):6580. doi: 10.3390/jcm11216580 (PMC9655254; doi:10.3390/jcm11216580)
Supplement: Supplementary file 1 [file jcm-11-06580-s001.zip › jcm-1841595-supplementary.pdf]

PRISMA 2020 flow diagram for new systematic reviews which included searches of databases and registers only

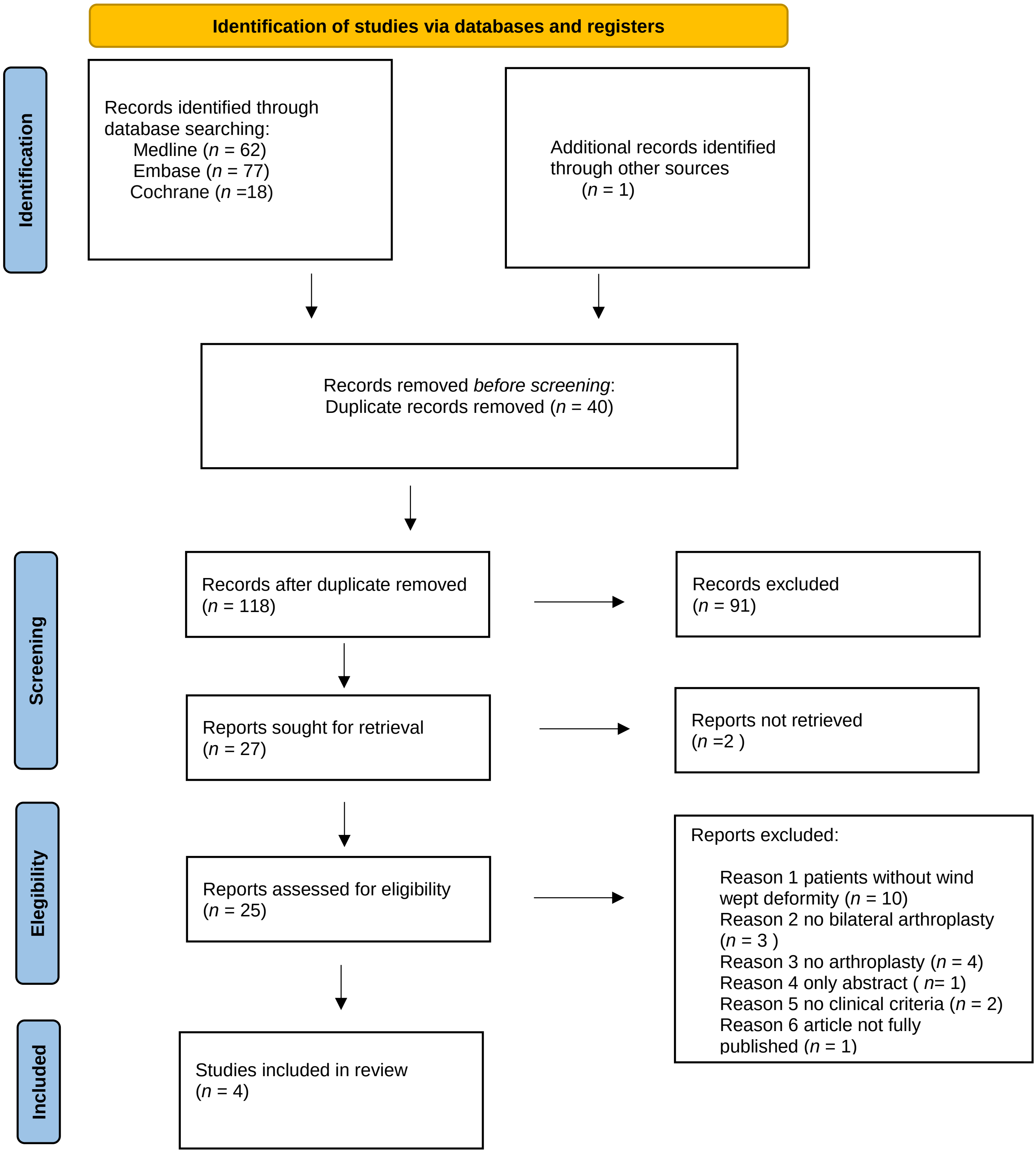

Figure S1. PRISMA Flowchart.

*Consider, if feasible to do so, reporting the number of records identified from each database or register searched (rather than the total number across all databases/registers).

**If automation tools were used, indicate how many records were excluded by a human and how many were excluded by automation tools.

PRISMA 2020 flow diagram for new systematic reviews which included searches of databases and registers only

From: Page MJ, McKenzie JE, Bossuyt PM, Boutron I, Hoffmann TC, Mulrow CD, et al. The PRISMA 2020 statement: an updated guideline for reporting systematic reviews. BMJ 2021;372:n71. doi: 10.1136/bmj.n71

For more information, visit: http://www.prisma-statement.org/
